# Family as a Pathway to Suicidal Behaviors Through Depression Symptoms and Internalized Homophobia

**DOI:** 10.1007/s40615-024-01956-8

**Published:** 2024-03-05

**Authors:** Donte T. Boyd, Emma Sterrett-Hong, Edward D. Scott, Junior L. Allen, Brianna Smith, Camille R. Quinn

**Affiliations:** 1 College of Social Work, The Ohio State University, Columbus, USA; 2 Center for Equitable, Family and Community Wellbeing, Ann Arbor, USA; 3 Raymond A. Kent School of Social Work and Family Science, University of Louisville, Louisville, USA; 4 Graduate College of Social Work, University of Houston, Houston, USA; 5 School of Social Work, Wayne State University, Detroit, USA; 6 School of Social Work, University of Michigan, Ann Arbor, USA

**Keywords:** Suicidal behaviors, Depression, Black MSM, Internalized homophobia

## Abstract

Research consistently highlights how systemic and social factors can adversely impact mental health, and the potential buffering effects of family support, yet raced sexual minorities are vastly underrepresented among these studies. As rates of suicide increase among Black people and remain high among men and those in gender and sexually diverse communities, this study sought to examine to relationships between family dynamics and suicidality among young Black men who have sex with men (MSM) in young adulthood. We used an online survey to conduct a logistic regression to examine family factors (family support, open family communication, other adult support, and other adult value), depression symptoms, and internalized homophobia on suicide attempts. The conceptualization of the study’s design and interpretation of the results were informed by minority stress theory and the phenomenological variant of ecological systems theory. The results indicate that higher levels of family support and open family communication were associated with lower levels of suicidality. Implications for future research and applications for healthcare providers and human services professionals who support young Black MSM in emerging adulthood are discussed.

## Introduction

Suicidality among young Black MSM is a potential epidemic that has been significantly understudied. Suicide among Black Americans in general, after decades of undercounting and misdiagnosis, has begun to be acknowledged as an important public health concern [1, 2]. While suicide rates among white Americans decreased between 2018 and 2021, they increased among many people of color, including Black Americans, who saw a 36.6% increase in completed suicides among those ages 10 to 24 and a 22.9% increase among those 25 to 44 years during that time [3]. Black American adults demonstrate the highest rates of suicidal ideation among adults seeking treatment at emergency departments, with 68 per 10,000 Black emergency department patients reporting suicidal ideation, compared to 42 of white, 25 of Hispanic/Latinx, and 19 of patients with other racial identities per 10, 000 endorsing suicidal ideation [4]. Moreover, in contrast to the general US population in which suicide deaths peak in the 45 to 54 years old age range, suicide rates for Black Americans peak in young adulthood. Among Black Americans ages 15 to 24 years old, 9.3 per 100,000 die by suicide, among Black Americans ages 25 to 34, 11.4 per 100,000 die by suicide, and among those ages 35 to 44, the rate is 9.1 per 100,000, with rates steadily decreasing for every age range thereafter [5]. These are likely underestimates as Black American who have died are more likely than white Americans who have died to have the cause of death classified as “undetermined,” due to less comprehensive and precise documentation of mental health concerns, among Black Americans than White Americans [6].

In addition, LGBTQ + adults across all races have reported higher rates of suicidality, including suicidal thoughts, plans, and attempts, than their cisgender heterosexual counterparts [7, 8]. Among young men ages 18 to 25, 18.4% of gay men and 22.2% of bisexual men have experienced suicidal ideation [6] compared to 6.7% of heterosexual men. In addition, 7.4% of gay and bisexual men ages 18 to 25 compared to 1.9% of their heterosexual counterparts have planned to attempt suicide, and 3.3% have attempted suicide [6] compared to 1.1% of heterosexual young men. Thus, it is critically important to examine precursors to suicidality among individuals at the intersection of these two marginalized identities, Black American and LGBTQ + .

The literature on suicidality among Black gay, bisexual, queer, and other sexual minority (GBQ +) men specifically is small but has begun to reveal some unique findings. Young Black GBQ + men experiencing greater structural racism at the state level, including residential segregation, adversely disparate incarceration rates and educational attainment, and higher levels of anti-LGBTQ state policies, were more likely to have attempted suicide [9]. However, minimal research has examined risk factors for suicidality at the individual and community levels.

The broader suicidality literature suggests intrapersonal and community factors that may act as risk factors for suicidality among young adults in general. Among emerging and young adults, depression has been linked to higher rates of suicidal ideation [10–12], suicide attempts [13], and completed suicides [14]. Relatedly, internalized homophobia has been examined in multiple studies with LGBTQ + young adults and has been identified as a risk factor for suicidal ideation [15] and suicide attempts [16].

Interpersonal factors also have been identified as significant catalysts for suicidality, particularly during emerging and young adulthood. The interpersonal theory of suicide postulates that disruptions in one’s sense of human connections, specifically the lack of social connections (thwarted belongingness) and perceived burdensomeness, are associated with suicidal ideation and behavior [17]. Family rejection and negative treatment by family members have also been linked to suicidal ideation [18] and suicidality among LGBTQ + young adults [19].

Conversely, across youth and young adults of diverse race/ethnicities and sexual identities, family cohesion predicts lower risk of suicidal ideation and suicide attempts [20, 21]. Closeness to family members has also been linked to lower levels of suicide ideation and attempts among Black American adults [19]. Connections to individuals in one’s community have been examined to a relatively lesser extent as a predictor of suicidality; however, neighborhood support during adolescence has been found to predict lower risk of suicidal ideation and suicide attempts in young adulthood among youth of diverse race/ethnicities and sexual identities [20, 21].

### Theoretical Framework

Minority stress theory (MST) is a primary theoretical framework employed in research focused on mental health disparities among sexual minorities [22, 23] and has been referenced in work conducted by the Centers for Disease Control and Prevention [22] and the Healthy People 2030 initiative of the US Department of Health and Human Services. MST posits that people from gender and sexually diverse (GSD) communities experience stress associated with their marginalized social status, which has an adverse impact on their health and well-being, particularly their mental health [23]. Thus, MST helps to conceptualize how the violence, discrimination, and victimization that people in GSD communities experience is an outgrowth of pervasive homophobia and consequently contributes to mental health disparities and psychological distress among sexual minority populations [24, 25].

Prior research has examined how the different facets of minority stress connect to suicide outcomes among sexual minority men [26, 27]. For instance, internalized homophobia or shame [28], family and peer rejection [29], becoming homeless after disclosure of sexuality [30], and perceived stigma [31, 32] were each found to be associated with suicidal ideation or suicide attempts among sexual minority men. Each of those facets reflects what MST scholars recognize as proximal and distal stressors [23]. MST suggests that whereas distal stressors are interpersonal and institutional demands (e.g., discriminatory policies or community violence) that compromise the well-being of GSD people, distal stressors are the internalized beliefs and value judgments resulting from socialization (e.g., internalized homophobia or feeling forced to conceal one’s sexuality). MST’s consideration of internalized, interpersonal, and institutional factors speaks to the role environment plays in sexual minority men’s experiences of minority stress. However, it also is helpful to consider how they strive to navigate and cope with that stress in context, as well as how contexts themselves can serve as stressors.

Thus, we additionally leverage the phenomenological variant of ecological systems theory (PVEST). PVEST calls for the consideration of ecosystem contexts, culture, and identity collectively with one’s experiences of stressors across the life course. PVEST also highlights the role of meaning making and acquired coping strategies in navigating those contexts [33, 34]. Black sexual minority men in emerging adulthood are challenged to navigate their early adult life amid anti-Black racism and homophobia. PVEST helps us conceptualize the particularity of their experience. PVEST invites us to examine how Black sexual minority men’s development and well-being is uniquely impacted by access to certain social supports and coping strategies, how various social contexts interact with them as Black sexual minority men, and their responses those environmental considerations. For example, whereas research generally asserts that familial support is a significant factor in how GSD people experience psychological health and stress [35–37], PVEST invites us to consider how culturally and contextually specific considerations are uniquely relevant to Black sexual minority men’s navigation of stressors and supports in their familial contexts [38, 39].

By leveraging MST and PVEST, we are better able to conceptualize how particular contexts and stressors contribute to suicidality among young Black MSM in emerging adulthood. MST helps conceptualize how the unique stressors relate to health-related outcomes, and PVEST provides a framework for considering the other contextual factors that influence that relationship. However, no study has examined the relationship among the family context, minority stress, and suicidal outcomes among young Black men who have sex with men, despite recognition that family rejection can contribute to suicidal outcomes among sexual minority males.

### Current Study

Black men, especially young Black MSM, are an understudied group in suicide research [40, 41]. Moreover, existing research does not reflect the nuances of within-group differences and tends to focus on homogeneous outcomes [42]. Very few studies focus on young Black MSM, even though they are at higher risk for suicide outcomes than their heterosexual peers [9]. This is one of the first studies to examine family dynamics and other adult support of young Black MSM ages 18 to 29 and the effect of these dynamics on suicidal behaviors. We apply the MST and PVEST to examine how risk and protective processes in the family context (including family bonding, open family communication, and other adult support) internalized homophobia, and planning to die by suicide, are associated with suicide attempts.

## Methods

### Recruitment and Procedures

This study used data from a larger study that examined strength-based approaches to sexual, physical, and mental health and suicidal behaviors among young Black men aged 18 to 29 who have sex with men [61]. The survey was programmed for different sampling sites, using Qualtrics software. An anonymous link was generated and included on a recruitment flyer. The flyer was then distributed via social media sites (e.g., Twitter and Facebook), and the link was to the survey was placed on Amazon Mechanical Turk (MTurk) [42]. To reach community-based organizations (CBOs)—that provides social services and other healthcare needs to lesbian, gay, bisexual, transgender, and queer individuals (LGBTQ +) individuals—the research team shared the flyer with community health workers at these types of CBOs, who then distributed the survey to eligible participants. The principal investigator and research assistants distributed the survey via social media every morning at 8 a.m. Our survey used Qualtrics bot protection and checked IP addresses to ensure that respondents were in the United States and to maintain data integrity by not allowing the same respondent to answer the eligibility or survey questions more than once. Participants who completed the survey and provided their email address received an electronic $35 Amazon gift card, with the exception of MTurk. Data was collected from social media sites, community-based organizations, and Amazon M-Turk for 60 days from December 1, 2021 to January 31, 2022.

Mechanical Turk provides a cost-effective and rapid method of recruitment for research studies that span multiple disciplines, including public health [43]. To view and participate in the survey, individuals who were registered with MTurk were required to have an approval rating of 95% or higher from previous surveys, be ages 18 or older, and reside in the United States (confirmed during the initial MTurk registration) [44, 45]. In addition, potential respondents who logged on to the MTurk platform during the week in which the survey was administered were informed that they had an opportunity to take a “survey about strength-based approaches to sexual, physical, and mental health and suicidal behaviors among young Black men who have sex with men aged 18 to 29.” Participants were notified that the survey would take 20 min. Participants were instructed to complete the survey in one sitting and were paid one dollar and given other incentives from MTurk [44, 45].

The inclusion/exclusion criteria were the same for all sampling sites. Respondents were eligible to participate in the study if they self-identified as Black or African American, were ages 18 to 29, resided in the United States, were assigned as male at birth, were fluent in English, currently identified as a man, and reported sexual contact (oral, anal, or otherwise) with a male in the previous year. We recruited a total of 400 Black gay and bisexual males ages 18 to 29. Most participants were recruited from MTurk (*n* = 200), followed by community-based organizations (*n* = 100), and social media sites (*n* = 100). After clicking on the survey link, participants were provided with an informed consent form and were asked to complete a screening tool to assess study eligibility. Individuals who met the inclusion criteria were asked a series of questions on demographics, mental health, and protective mechanisms. Participants who used social media sites and MTurk to complete the survey used their own computers. Individuals who completed the survey in a community-based organization used a computer or tablet provided by the organization.

### Participants

The majority of the sample identified as Black American or African American (75%), followed by Caribbean (10%), Afro-Latino (10%), and continental African (5%). Thirty percent of the sample never attended high school and 29% had completed college or postgraduate studies. The average household income ranged from less than $20,000 to $150,000, with the average household income being $57,499. Most of the sample (95%) reported being assigned male at birth, and 5% were assigned female. Transmen were encouraged to participate in the study. All participants self-reported having sex with men within the last year. Forty-five percent of the sample reported being gay, 35% straight or heterosexual, 10% bisexual, 5% questioning, and 5% other.

### Measures

#### Outcome Variable: Past-Year Suicide Attempt

We assessed suicide attempts using a single item that asked respondents to indicate whether they had attempted to end their life within the previous 12 months. Response categories were 1 (*yes*) and 0 (*no*) [46]. Twenty-seven percent of participants had attempted suicide in the past year.

#### Independent Variables

##### Planning to Die by Suicide in the Past Year

We measured suicide planning with a single item that asked participants whether they had made a plan to end their life within the previous 12 months. Response categories were 1 (*yes*) and 0 (*no*) [46]. Thirty-three percent of participants had made a plan to end their life in the previous 12 months.

##### *Depression Symptoms*

We used the Center for Epidemiological Studies Depression Scale (CESD-10) to measure depression symptoms [47]. The CESD-10 assesses depressive symptoms experienced in the past week. Prior research has validated the measure among clinically depressed populations, the general population, and sexual minorities of color [10]. Sample items included “How many times in the past week did you feel as good as other people?” and “How many times in the past week did you have trouble keeping your mind on task?” Response options range from zero (*Rarely or never*) to 3 (*Most or all of the time*). The CESD-10 scores range from zero to 30, with higher scores indicating more depressive symptoms (Cronbach’s *α* = 0.81). Individuals with scores above 20 were classified as having moderate to severe depression symptoms [48].

##### *Internalized Homophobia*

Internalized homophobia was measured using nine items on a 5-point Likert scale ranging from 1 (*Strongly disagree*) to 5 (*Strongly agree*) [49]. This scale assessed the extent to which lesbian, gay, and bisexual individuals reject their sexual orientation, are uneasy about their same-sex desires, and seek to avoid same-sex attractions and sexual feelings. Sample items include “I often feel it best to avoid personal or social involvement with other gay/bisexual men” and “I feel alienated from myself because of being gay/bisexual.” For the current study, a total score was tabulated by summing all items and the Cronbach’s *α* = 0.96.

The authors used validated measures for each construct. *Family support* was measured using three items on a 5-point Likert scale ranging from 1 (*strongly disagree*) to 5 (*strongly agree*). Participants were asked questions such as “My parents give me help and support when I need it.” We averaged responses to these three items, with higher scores indicating more family support [50]. The Cronbach’s *α* = 0.95. *Open family communication* using a single item on a 5-point Likert scale ranging from 1 (*strongly disagree*) to 5 (*strongly agree*). Participants were asked to rate the statement “I have lots of good conversations with my parents” [50]. *Other adult values* was measured using a single 5-point Likert-type question, with values ranging from 1 (*strongly disagree*) to 5 (*strongly agree*), asking participants to rate the statement “Adults in my town or city listen to what I have to say,” with a higher score indicating that adults in the community listened to the young men [50]. *Other adult support* was measured by using a single 5-point Likert-type question, with values ranging from 1 (*strongly disagree*) to 5 (*strongly agree*), asking participants to rate the statement “Adults in my town or city make me feel important,” with a higher score indicating that adults in the community supported the young men [50].

Several contextual variables were collected. Participants were asked to indicate their education, age, employment status, and household income. Laid off due to COVID-19 was measure using a single, asking participants were they laid off because corona virus, responses ranged from 1 (yes), 2 (no), I was laid off for other reasons, 3 (not applicable), and I was not working prior to COVID-19. Ethnicity was measured using a single item, asking participants their ethnicity, ranging from 1 (Black American), 2 (Caribbean (e.g., Jamaican, Haitian)), 3 (Continental African (e.g., Nigerian)), and (Afro-Latino (e.g., Dominican)).

### Statistical Analysis

Table 1 presents descriptive statistics of study variables. We conducted a bivariate regression analysis between independent variables and the outcome variable, suicide attempts (Table 2). Our team utilized STATA 18 to conduct a logistic regression analysis examining whether family factors (family support, open family communication, other adult supports, and other adult values) depression symptoms, internalized homophobia, on suicide attempts (Table 3). We conducted a logistic regression because the dependent variable was binary, suicide attempts. The percentage of missing data was less than 5%.

**Table 1:** Demographics and sample characteristics (*N* = 400)

Variable	*M* or %	*SD* or *N*	Range (if applicable)
Age	23.46	2.59	18–29
Household income	$57,499.50	1.34	Up to $150,000
Race or ethnicity			
Black American or African American	75%	300	
Caribbean (e.g., Haitian or Jamaican)	10%	40	
Continental African (e.g., Ghanaian or Nigerian)	5%	20	
Afro-Latino (e.g., Dominican)	10%	40	
Sex assigned at birth			
Male	95%	380	
Transgender male	5%	20	
Sexual orientation			
Heterosexual or straight	35%	140	
Gay	45%	180	
Bisexual	10%	40	
Questioning	5%	20	
Other	5%	20	
Education			
Never attended school	30%	100	
Less than high school	19%	65	
Some high school	4%	12	
High school diploma or GED	2.4%	8	
Some college, associate degree	14%	47	
College, postgraduate	29%	98	
Currently in school	1.5%	5	
Depressive symptoms	14.46%	5.97	
Laid off due to COVID-19			
Yes	83%	250	
No, laid off for other reasons	14%	50	
Not applicable; not working prior	14%	50	
Internalize homophobia	3.0	1.24	1–5
Family support	3.84	0.93	1–5
Open family communication	3.88	1.03	1–5
Planned to die by suicide in the past year			
Yes	33%	130	
No	66%	220	
Suicide attempt			
Yes	27%	98	
No	72%	252

**Table 2:** Bivariate regression analysis of key study variables (*N* = 400)

Variables	OR	SE	95%CI
Suicide attempts			
Planned to die by suicide in the past year	22.76***	7.40	12.02, 43.07
Depression symptoms	1.19***	0.03	1.13, 1.26
Internalize homophobia	2.52***	.0.35	1.91, 3.31
Family support	0.95	0.04	0.87, 1.03
Open family communication	1.01	0.12	0.79, 1.29
Other adult values	1.29*	0.19	1.02, 1.73
Other adult supports	1.01	0.12	0.79, 1.28
Laid off due to COVID-19			
No, I was laid off for other reasons (reference)			
Yes	1.96	0.81	0.86, 4.44
Not applicable, I was not working prior to COVID-19	0.73	0.53	0.17, 3.08
Employment			
Full time (reference)			
Self-employed	0.88	0.43	0.33, 2.29
A homemaker	1.28	0.65	0.47, 3.51
Unable to work (disabled)	0.46	0.50	0.05, 3.93
Education			
Never attended school (reference)			
Less than high school	0.23***	0.09	0.10, 0.49
Some high school	1.06	0.65	0.32, 3.56
High school diploma or GED	0.64	0.48	0.14, 2.84
Some college, associate degree	0.34**	0.14	0.15, 0.76
College, post-graduate	0.13**	0.05	0.06, 0.28
Currently in school	0.35	0.41	0.03, 3.55
Age	1.04	0.05	0.95, 1.15
Household income	0.77***	0.07	0.63, 0.93
Ethnicity			
Black American			
Caribbean (e.g., Jamaican, Haitian)	1.26	0.70	0.42, 3.77
Continental African (e.g., Ghanaian or Nigerian)	1.86	1.71	0.30, 11.35
Afro-Latino (e.g., Dominican)	1.39	0.87	0.41, 4.76

*p* < .05*, *p* < .01**, *p* < .001***; not employed had less than 5 cases and so was not used in the analysis

*OR* odds ratios, *SE* standard errors, *CI* confidence intervals

**Table 3:** Logistic regression analysis on suicide attempts among young Black MSM (*N* = 400)

	AOR	SE	95% CI
Suicide attempts			
Planned to die by suicide in the past year	11.31***	4.25	5.42, 23.61
Depression symptoms	2.00***	0.04	1.06, 3.83
Internalize homophobia	2.32**	0.69	1.29, 4.15
Family support	0.41**	0.13	0.21, 0.78
Open family communication	0.30***	0.22	0.06 0.73
Other adult values	1.50*	0..29	1.02, 2.20
Other adult supports	0.94	0.15	0.67, 1.30
Laid off due to COVID-19			
No, I was laid off for other reasons (reference)			
Yes	2.79**	1.36	1.07, 72.5
Not applicable, I was not working prior to COVID-19	0.73	0.58	0.15, 3.47
Employment			
Full time (reference)			
Self-employed	0.48	0.29	0.14, 1.60
A homemaker	0.67	0.43	0.19, 2.34
Unable to work (disabled)	0.26	0.30	0.02, 2.60
Education			
Never attended school (reference)			
Less than high school diploma	0.20**	0.11	0.09, 0.59
Some high school	1.36	1.01	0.31, 5.87
High school diploma or GED	0.64	0.52	0.13, 3.20
Some college, associate degree	0.29**	0.13	0.12,0.69
College, post-graduate	0.11**	0.05	0.05, 0.27
Currently in school	0.26	0.31	0.02, 2.77
Age	1.16**	0.07	1.03, 1.30
Household income	0.89	0.11	0.70, 1.15
Ethnicity			
Black American			
Caribbean (e.g., Jamaican, Haitian)	1.02	0.73	0.25, 4.13
Continental African (e.g., Ghanaian or Nigerian)	0.33	0.42	0.03, 4.09
Afro-Latino (e.g., Dominican)	2.06	1.51	0.50, 8.70

*p* < .05*, *p* < .01**, *p* < .001***; not employed had less than 5 cases and so was not used in the analysis

*AOR* adjusted odds ratios, *SE* standard errors, *CI* confidence interval

## Results

Twenty-seven percent of Black men who have sex with men (BMSM) reported that they had attempted suicide in the past year, and 33% of the sample reported that they had planned to die by suicide in the past year. BMSM reported moderate forms of internalized homophobia (*M* = 3.0; *SD* = 1.24). Family bonding (*M* = 3.84; *SD* = 0.93) and open family communication (*M* = 3.84; *SD* = 1.03) among BMSM were slightly high (see Table 1). Bivariate regression (see Table 2) indicated planning to die by suicide was positively associated with suicide attempts (OR: 22.76; 95% confidence interval [CI]: 12.02, 43.07]. Black MSM who experienced depression symptoms were 1.19 times more likely to attempt suicide than those who did not experience depression symptoms (OR:1.19; 95%CI: 1.13, 1.26). Black MSM were 2.52 times more likely to attempt suicide if they experienced internalized homophobia (OR: 2.52; 95%CI: 1.91, 3.31).

### Logistic Regression

The logistic regression analysis (see Table 3) examines the direct effects from family support, open family communication, other adult supports, other adult values, depression symptoms, internalize homophobia, and suicide planning on suicide attempts. Planning to die by suicide was positively associated with suicide attempts (OR: 11.31; 95% confidence interval [CI]: 5.42, 23.61]. Black MSM who experienced depression symptoms were 2 times more likely to attempt suicide than those who did not experience depression symptoms (OR: 2.00; 95%CI: 1.06, 3.83). Black MSM were 2.32 times more likely to attempt suicide if they experienced internalized homophobia (OR: 2.32; 95%CI: 1.29, 4.15). Family support (OR: 0.41; 95%CI: 0.21, 0.78) and open family communication (OR: 0.30; 95%CI: 0.06, 0.73) were associated lower odds of attempting suicide. Other adult support was associated with an increase in internalized (OR: 1.50; 95%CI: 1.02, 2.20). Black males who self-reported being laid off from employment because of COVID-19 were almost 3 times more likely to attempt suicide than those who were not laid off (OR: 2.79; 95%CI: 1.07, 72.5). Black males who self-reported having less than high school diploma (OR: 0.20; 95%CI: 0.09, 0.59), some college, associate degree (OR: 0.29; 95%CI: 0.12, 0.69), and college and postgraduate (OR: 0.11; 95%CI: 0.05, 0.27) were all less likely to commit suicide than those who never attended school. Older Black men were more likely to attempt suicide than younger males (OR: 1.16; 95%CI: 1.03, 1.30).

## Discussion

This is one of the first studies to examine the influence of family factors including other adult support and value, internalize homophobia, depression symptoms planned to die by suicide in the past year on suicide attempts among young Black MSM (18 to 29). It is important to understand the nuance in relationships between Black families and their sons, as they play a critical role in influencing their mental health. For instance, prior research has indicated that family support and cohesion was associated with lower risk of suicidal ideation and attempts [20, 21]. In other research, guided by MST has indicated that family rejection is considered a stressor that has been associated with internalized homophobia, depression, and elevated suicide risk [22, 23]. However, because of the scant research on Black family relationships with their sexual gender minority sons and their influence on suicidal behaviors, an investigation is warranted.

This study was guided by the MST and the PVEST model to better understand how the Black family context and stressors (e.g., internalize homonegativity, discrimination) influence young Black MSM suicidal behaviors in emerging adulthood. This is critical because there are high rates of suicide planning (33%) and attempts (27%) found among participants in this study, compared to rates among MSM across races/ethnicities of suicide planning (7.4%) and attempts (3.3%) found in a recent large nationally representative sample, revealing the urgent need to attend to and address mental health and suicidality among young Black MSM. Importantly, these rates are somewhat higher than those reported in a recent study using data from the National Survey of Drug Use and Health (NSDUH), which included nearly 192,000 participants, 14,693 of whom identified as lesbian, gay, and bisexual (LGB) [49]. In that study, 4% of Black gay and bisexual men reported suicide plans and 2.6% reported suicide attempts. The higher rates in the current study may be related to the recruitment of young adult men specifically and are perhaps also due to the somewhat more anonymous nature of data collection through online survey platforms compared to in-person interviews, as were used in NSDUH. Further investigations to determine what family factors underlie these increases are necessary to provide avenues for interventions needed to mitigate the likelihood of these behaviors.

Our results indicated that higher levels of family support were associated with lower levels of suicide attempts. These findings are consistent with prior literature that an increase in family support is associated with lower suicide ideation [12] among Black youth and emerging adults including young Black MSM. In addition, respondents’ report of parental support, as compared to the level of communication, may better indicate greater parental acceptance of participants in a holistic sense, including sexuality identity. Parental acceptance of sexual identity has been linked to more positive mental health indicators [51], providing support for the findings in the current study. This study extends the current literature, as our results indicated parental support was associated with lower likelihood of young Black MSM attempting suicide. Our results have implications for family interventions and practices, as positive parent support experiences between Black parents and their sons allows for positive coping, loving, and secure relationships across the life span, which is critical for this population due to the external stressors they face.

Our results indicated that more positive family communication was associated with lower levels of suicidality. This is consistent with prior studies that have found LGBTQ + young adults to report positive relationships with families prior to coming out, which continued after their disclosure [52]. Other studies have indicated that positive family communication has a positive influence on health-related outcomes and this study includes suicide attempts among young Black MSM to address a key gap in the literature. Participants reporting good communication may also have decided to share their sexual identity with their families, which has been related to higher rates of self-acceptance.

Similarly, greater endorsement of being valued by one’s town or city was linked to higher levels of suicide attempts. Some recent work has suggested that to some extent Black LGBTQ + adolescents are more likely than their white counterparts to report lower levels of “outness” (such as being mostly not out) [39]. Therefore, youth and young adults could feel valued by adults in their town or city, but that feeling of being valued may come at the cost of feeling less safe to be their authentic selves.

Our findings indicated that among young Black MSM planned to die by suicide, internalized homophobia and depression symptoms were directly associated with suicide attempts. Research has indicated that internalized homophobia and depression leads to poor mental health outcomes [53] and that depression was higher among sexual gender minorities than their heterosexual peers [54]. These studies also showed that parent rejection leads to internalized homophobia and depression [53, 54]. Furthermore, being young, Black, and self-identifying as MSM critically impacts suicidal thoughts, plannings, and attempts. These findings indicate a strong need for social workers and other practitioners to implement and adopt an intersectional approach when looking at improving mental health outcomes of young Black MSM especially in the context of family.

The high rates of planning to die by suicide and attempts in this study are particularly salient, given recent findings that LGTBQ + youth experiencing higher levels of minority stress are also more likely to disclose their suicidal ideation [15]. In addition, Black youth in general and young adults, regardless of sexual orientation, who have attempted suicide are less likely than white youth and young adults who have attempted suicide to have reported suicidal ideation [55], equating to less opportunity to intervene to reduce the risk of suicidality among Black youth and young adults. Educating parents, family members, and other supportive adults of young Black MSM on how to respond to a suicide crisis may provide an opportunity for timely intervention if young Black men are experiencing a mental health crisis.

 Our results indicated that young Black men who were laid off because the COVID-19 pandemic were more likely to attempt suicide. This is consistent with prior literature that depression and anxiety increased during the pandemic among sexual gender minorities [56]. Sexual gender minorities are more likely to experience lack of employment and being unhoused in comparison to their heterosexual counterparts [57]. It is possible that COVID-19 exacerbated these unemployment rates among young Black MSM that led them to think, plan, and attempt suicide.

Our results also revealed that any level of education lowered suicide attempts of young Black MSM. The data was collected post COVID-19, and this demographic of young Black MSM may have experienced a heightened sense of belonging and connection because of their life experiences that had a positive influence on their mental health and suicidality. Education is a social determinant of health and plays a significant role in influencing an individual’s overall well-being, including suicidal behavior, i.e., suicide attempts [58], as well as their access to mental health support and the potential for increased socioeconomic status. Some scholars note that social support can promote feelings of a sense of belonging and reduce burdensome feelings through improving their sense of self-worth [59]. Further, Black MSM with higher levels of education have experienced lower rates of suicide attempts that can serve as a buffer or protective factor for them, especially if they did not have coinciding experiences of discrimination [12, 42]. Specifically, education can provide a vehicle for increased knowledge, development of critical thinking skills, as well as the ability to successfully navigate social and systemic barriers they may face due to their expanded social support networks. For this sample of Black MSM, they may have benefitted from the socialization school provided, especially post COVID-19. Given the strains and stress associated with the pandemic and its impact on their education, i.e., forcible move to online learning at home versus instruction in school settings, the social interaction in school may have enhanced their ability to cope with stress, discrimination, sexuality, and mental health challenges that reduced the likelihood of suicidal behavior. Another study suggested the adolescents in school were more likely to report suicidal behavior if they experienced teacher and peer-based discrimination [60]. Consequently, these factors may be less salient to young adult Black MSM.

We also found that older Black MSM were more likely to attempt suicide than younger Black MSM. Consistent with prior literature age can play a significant role in influencing suicide rates which includes young Black young men who have sex with men [61]. It is critical to understand that suicide is a complex issue and influenced by various factors, but understanding the impact of age can provide valuable insights for this population. During adolescence and early adulthood, individuals undergo significant physical, emotional, sexual, and social changes [61]. For young Black MSM, these transitional years can be particularly challenging, as they navigate the complexities of racial identity, discrimination, and socioeconomic disparities [60, 61]. These factors, combined with limited access to mental health resources and stigma surrounding mental health in certain communities, can contribute to increased vulnerability to suicide. These alarming rates can be attributed to a combination of factors related to age and the unique challenges faced by young Black men.

### Limitations

There are several limitations that should be noted in this study. First, this a cross-sectional study, and we cannot determine whether family factors such as bonding and open family communication are the direct cause of Black MSM depression and internalized homophobia as well as contributing indirectly to suicidal behaviors. Prior longitudinal research has indicated that family risk often predates mental health symptoms [55], but these associations are also likely to be bidirectional, and this study examines the strengths of families. Similarly, indirect effects suggest potential mediating pathways of family factors on suicidal behaviors through depressive symptoms and internalized homophobia, but these should not be interpreted causally. This study also used two measures as proxies for other adult support among Black MSM. Future research should use a validated scale to fully understand the role of other adult support for young Black MSM. Lastly, given the complex and multifactorial causes of suicidal behaviors, it is crucial to understand how family dynamics and relationships influence the suicide risk of Black MSM.

### Implications and Future Directions

Results of this study have implications for future research, as well as the practice of healthcare providers and human services professionals who support young adult Black MSM. Of note is the prevalence of high suicidality among the participants. The high rates of suicide planning and attempts in this study are particularly salient given recent findings that LGTBQ + youth experience higher levels of minority stress [15]. Researchers must seek to identify the factors that most effectively reduce suicidality among young Black MSM considering the potentialities for cultural and ecosystemic factors to serve as buffers against suicide, particularly within the family context. Future research should investigate further what is “good family communication” between young Black MSM and their families. In addition, the context of these discussions and how they influence suicide behaviors. Healthcare and human service providers, particularly those providing mental health counseling services and family-based interventions, are well positioned to capture preliminary data and contribute to practice-informed research that leads to identifying or developing pragmatic support strategies. These study results also suggest the utility of empirically examining how Black gay and bisexual men discuss or desire to discuss mental health and identity in their families. Such follow-up studies must account for how homophobia and mental health stigma (ableism) influence the existence, content, and experience of those conversations.

Additionally, a recent systematic review elucidated the unique role of chosen families or fictive kinship networks as a protective factor for Black LGBTQ + people [57]. Therefore, whether the relationship quality and dynamics with chosen family members or other adult support are associated with suicidality deserves additional research attention. This information may be particularly useful for social workers and human service professionals assisting Black gay and bisexual youth who are establishing kinship care and social support networks.

Lastly, future research also should examine the extent to which racial discrimination is linked to suicidality among young Black MSM. In addition to homophobia and mental health stigma, anti-Black racism is a social reality with which young Black MSM contend. A recent study found that police-involved killings of Black Americans within the past month are associated with higher rates of completed suicides among Black Americans [58]. This prompts a question about how these killings and other racial violence might be uniquely experienced by young Black MSM situated at the same social location. One cannot help but consider the recent killing of O’Shea Sibley, and the unique impact his death has on the psychological well-being and future outlook of young Black MSM who might see themselves in his story. Mental health clinicians must be aware of how these events can be unique potential stressors for Black gay and bisexual young men and leverage insights from this research and future studies to provide effective, culturally responsive care. Further, research that investigates the roles of protective factors like social support and academic attainment may serve as the key vehicles to explore gender and LGBTQ specific characteristics of Black children, including young Black MSM with suicide ideation to provide evidence that can be used to develop gender and racial specific interventions for them [59].

## Conclusion

Family factors have been linked to youth depressive symptoms, internalized homophobia, and suicidal behaviors, but limited research has focused on young Black men who have sex with men (MSM). It is crucial to explore the family dynamics of young Black MSM to understand how these factors impact their mental health and well-being. Additionally, it is essential to view the family as a source of strength and resilience for these individuals. Previous studies have shown that supportive families can serve as a protective factor, reducing depression and suicide risk among Black youth.

Developing a culturally sensitive family-based intervention tailored to the unique needs of young Black MSM is vital in addressing mental health disparities. This study represents one of the initial efforts to investigate the family context of Black MSM in relation to internalized homophobia, depression, and suicidal behaviors. Future research should delve deeper into the intersectionality of being a Black MSM within the family context and how these different aspects of identity influence suicidal behaviors. By understanding and leveraging the potential strengths of family support, interventions can be designed to promote mental health and well-being among young Black MSM, ultimately working towards reducing racial disparities in mental health outcomes.

## Data Availability

The data produced in this research project is available upon request from the lead author.
